# DNA hypomethylation at specific CG-sites within *TRAK1* is linked to the neurocognitive profile in Klinefelter syndrome

**DOI:** 10.1038/s41380-025-03254-z

**Published:** 2025-09-30

**Authors:** Helene B. L. Tallaksen, Emma B. Hasselholm, Joel B. Berletch, Gala N. Filippova, Xinxian Deng, Daniel L. Van Dyke, James W. MacDonald, Theo K. Bammler, Simon Chang, Cecilie D. R. Buskbjerg, Claus H. Gravholt, Christine M. Disteche, Jesper Just, Anne Skakkebæk

**Affiliations:** 1 Department of Molecular Medicine, Aarhus University Hospital, Aarhus, Denmark; 2 Department of Molecular Biology and Genetics, Aarhus University, Aarhus, Denmark; 3 Department of Clinical Medicine, Aarhus University Hospital, Aarhus, Denmark; 4 Department of Laboratory Medicine and Pathology, School of Medicine, University of Washington, Seattle, USA; 5 Mayo Clinic College of Medicine, Rochester, Minnesota USA; 6 Department of Environmental and Occupational Health Sciences, University of Washington, Seattle, WA USA; 7 Unit for Thrombosis Research, University Hospital of Southern Denmark, Esbjerg, Denmark; 8 Department of Endocrinology, Aarhus University Hospital, Aarhus, Denmark; 9 Department of Psychology and Behavioral Sciences, Aarhus University, Aarhus, Denmark; 10 Department of Medicine, School of Medicine, University of Washington, Seattle, USA; 11 Department of Clinical Genetics, Aarhus University Hospital, Aarhus, Denmark

**Keywords:** Genetics, Stem cells, Biomarkers

## Abstract

Klinefelter syndrome (47,XXY; KS) impacts neurodevelopment. Furthermore, KS is associated with widespread alterations in the epigenome and transcriptome. Whether these epigenetic and transcriptomic alterations can be linked to the neurocognitive phenotype remains to be elucidated. We performed a comprehensive, integrative analysis of the neurocognitive profile and the methylome in blood from males with KS (n = 65) and male controls (n = 63) (Cohort 1). The results were validated in a second cohort of males with KS (n = 22) and male controls (n = 16) in which transcriptome data was also available (Cohort 2). The findings were further validated in neural precursor cells derived from human induced pluripotent stem cells from 47,XXY (n = 3) and 46,XY (n = 3) amniotic cells. In cohort 1, we identified five CG-sites within the *TRAK1* gene which were hypomethylated in males with KS compared to male controls. *TRAK1* hypomethylation was positively correlated with several neurocognitive variables among males with KS. In cohort 2, we identified a similar methylation pattern and demonstrated that the methylation levels at the five CG-sites were correlated with a high expression level of a specific short *TRAK1* transcript (ENST00000341421.7). Neural precursor cells (NPCs) established from 47,XXY amniotic cells also exhibited hypomethylation at the five CG-sites and strong upregulation of ENST00000341421.7 compared to NPCs established from 46,XY amniotic cells. In conclusion, we demonstrate that the DNA methylation level at specific CG-sites within *TRAK1*, a gene highly expressed in the brain, is correlated with the neurocognitive phenotype of KS, implying a possible epigenetic underpinning for the observed neurocognitive impairments in KS.

## Introduction

Klinefelter syndrome (47,XXY; KS) is the most prevalent form of sex chromosome aneuploidy, affecting one in 660 males [1]. The phenotypic traits of KS include hypergonadotropic hypogonadism, small testes, infertility, altered body composition, and increased height (reviewed in [2]). KS also affects neurodevelopment and is associated with an increased risk of neurocognitive impairments, although the variability is large. Approximately 70–80% of males with KS have verbal deficits that affect both receptive and expressive language functions [3, 4]. Several studies [5–8] have also documented an increased frequency of impaired executive functioning in males with KS, including deficits in attention, working memory, cognitive flexibility, and inhibitory control. On average, full-scale IQ (FSIQ) of males with KS is about 10 points lower than that of the general population, with verbal IQ (VIQ) being more severely affected than performance IQ (PIQ) [5, 6, 8–11], which has been coined as an IQ split. This is also observed in Turner syndrome (45,X), but with a mirror-image of higher VIQ than PIQ [12].

Interestingly, a mirror-image of the DNA methylation pattern is also seen in KS and TS [13–15]. DNA methylation is crucial for normal brain development such as neuronal differentiation and maturation, and normal brain functions, including learning and memory [16–18]. Global cognitive ability and executive function have been reported to be associated with specific DNA methylation patterns [19]. We, and others, have previously shown that KS is associated with genome-wide alterations in DNA methylation across different tissues [13–15, 20–22]. Genes that are differentially methylated in KS are involved in a variety of biological processes related to neural development and neurotransmission, suggesting that alterations in DNA methylation could be implicated in the neurocognitive impairments seen in KS [14, 15].

Here, we present a comprehensive and integrative analysis of the neurocognitive profile and the methylome in KS, aiming to investigate the link between specific neurocognitive traits (processing speed, working memory, visual performance, verbal memory and learning, verbal fluency and comprehension, verbal performance, and executive functions) and DNA methylation patterns, and to identify potential epigenetic biomarkers of neurocognitive function in KS.

## Materials and Methods

### Participants

#### Cohort 1

**Fig. 1:**
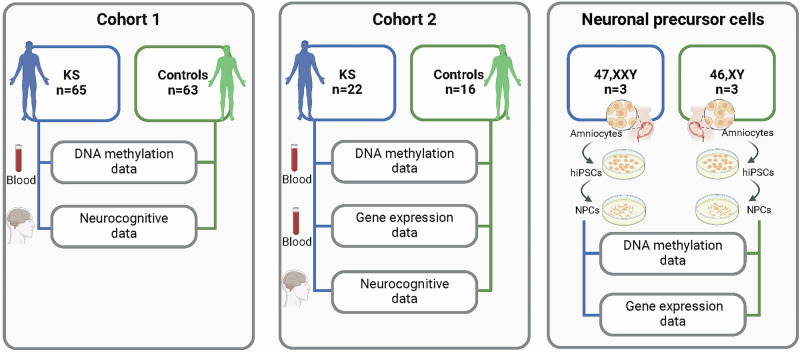
Overview of the cohorts, cells, tissue types and data included in the study. DNA methylation data from peripheral blood samples and neurocognitive data were investigated for cohort 1 (left panel) that included 65 males with KS and 63 male controls. For cohort 2 (middle panel; 22 males with KS and 16 male controls), DNA methylation and RNA sequencing data from peripheral blood samples as well as neurocognitive data were examined. DNA methylation and RNA sequencing data were investigated in XXY (n = 3) and XY (n = 3) neural precursor cells (right panel) derived from human induced pluripotent stem cells (hiPSCs), obtained from amniocytes. This figure was made using BioRender.com.

Males with verified KS and male controls were included as described previously [6, 14]. Participants were enrolled between 2009 and 2012. Neurocognitive data and DNA methylation data were available for 65 males with KS (mean age 36.8 ± 10.5 (SD) years) and 63 controls matched for age and education (mean age 36.4 ± 10.3 (SD) years) (Fig. 1; Table 1).

**Table 1:** Neurocognitive traits and age in men with Klinefelter syndrome (KS) and controls from cohort 1.

	KS (n = 45)	Controls (n = 43)	p-value KS vs. controls
Age (yr)	36.8 ± 10.5	36.4 ± 10.3	0.83
*Intelligence*			
FSIQ	87.8 ± 12.0	104.0 ± 11.4	<0.01
PIQ	96.7 ± 13.2	106.0 ± 10.8	<0.01
VIQ	84.7 ± 12.3	101.0 ± 11.8	<0.01
*Processing speed*			
WAIS-C	57 (31–91)	71 (39–112)	<0.01
TMT-A	29 (16–80)	23 (12–64)	<0.01
TMT-B	77 (25–205)	62 (32–208)	<0.01
*Working memory*			
WAIS-DS	13 (8–22)	15 (10–24)	<0.01
WAIS-LN	9 (4–18)	11 (7–18)	<0.01
*Visual performance*			
WAIS-BD	43 (19–66)	55 (12–66)	<0.01
WAIS-MR	18 (5–25)	21 (7–25)	<0.01
*Verbal memory and learning*			
RAVL-tot	43 (21–66)	51 (29–70)	<0.01
*Verbal fluency and comprehension*			
SR	15 (10–21)	17.5 (13–22)	<0.01
*Verbal performance*			
WAIS-V	24.5 (8–49)	37 (12–57)	<0.01
WAIS-S	17.5 (8–26)	23 (11–31)	<0.01
Fluency	51 (21–94)	67 (35–107)	<0.01
*Executive function*			
WCST-trai	113 (70–128)	83 (70–128)	<0.01
TOL-tms	26 (0–67)	15 (0–55)	<0.01
TOL-tcs	5 (0–10)	7 (1–10)	<0.01

Data are mean ± SD or median (total range). T-test or Wilcoxon test were performed according to distribution.

*FSIQ* full-scale IQ, *VIQ* verbal IQ, *PIQ* performance IQ, *WAIS-III* the coding subtest (WAIS-C) of Wechsler Adult Intelligence Scale III, *TMT-A* trail making a, *TMT-B* trail making B, *WAIS-DS* digit span subtests of WAIS-III, *WAIS-LN* letter-number sequencing subtests of WAIS-III, *WAIS-BD* block design subtests of WAIS-III, *WAIS-MR* matrix reasoning subtests of WAIS-III, *RAVL-tot* total score of rey auditory verbal learning test, *SR* sentence repetition, *WAIS-S* similarities subtests of WAIS-III and *WAIS-V* vocabulary subtests of WAIS-III, *Fluency* Verbal fluency test, *WCST-trai* cards administered of the Wisconsin Card Sorting Test, *TOL-tms* total move score, *TOL-tcs* total correct score of the Tower of London.

#### Cohort 2

Cohort 2 consisted of 22 males with verified KS (mean age 38.3 ± 8.1 (SD) years) and 16 male controls matched for age and education (mean age 38.4 ± 12.0 (SD) years) [15]. Participants were enrolled between 2015 and 2019. Males with KS were recruited from fertility and endocrinology clinics across Denmark. Controls were included by public advertising. DNA methylation data, gene expression data and neurocognitive data were available for all participants (Fig. 1; Table 2).

**Table 2:** Neurocognitive traits and age in men with Klinefelter syndrome (KS) and controls from cohort 2.

	KS (n = 22)	Controls (n = 16)	p-value KS vs. controls
Age (yr)	38.3 ± 8.1	38.4 ± 12.0	0.96
*Intelligence*			
FSIQ	92.9 ± 12.6	100.4 ± 9.4	0.04
PIQ	101.1 ± 16.5	100.5 ± 8.2	0.89
VIQ	86.5 ± 11.5	101.2 ± 13.9	<0.01
*Processing speed*			
WAIS-C	56.5 ± 12.3	59.1 ± 16.7	0.61
*Working memory*			
WAIS-DS	14.2 ± 3.3	16.8 ± 4.0	0.04
WAIS-LN	17.8 ± 3.0	18.9 ± 2.7	0.24
*Visual performance*			
WAIS-BD	52.0 (30–62)	48.5 (29–55)	0.40
WAIS-MR	16.0 ± 4.8	17.1 ± 3.9	0.41
*Verbal performance*			
WAIS-V	26.6 ± 6.5	32.3 ± 7.4	0.02
WAIS-S	18.0 ± 4.4	22.9 ± 4.5	<0.01
*Executive function*			
WCST-trai^1^	109.0 (74–128)	94.0 (73–128)	0.21

Data are mean ± SD or median (total range). T-test or Wilcoxon test were performed according to distribution. ^1^Only 15 controls were included.

*FSIQ* full-scale IQ, *VIQ* verbal IQ, *PIQ* performance IQ, *WAIS-III* the coding subtest (WAIS-C) of Wechsler Adult Intelligence Scale III, *WAIS-DS* digit span subtests of WAIS-III, *WAIS-LN* letter-number sequencing subtests of WAIS-III, *WAIS-BD* block design subtests of WAIS-III, *WAIS-MR* matrix reasoning subtests of WAIS-III, *WAIS-S* similarities subtests of WAIS-III, *WAIS-V* vocabulary subtests of WAIS-III, *WCST-trai* cards administered of the Wisconsin Card Sorting Test.

### Ethics

The study was approved by The Danish Data Protection Agency and the local ethics committee (Region Midtjylland, Denmark number M-20080238, Central Denmark Regional Committee on Health Research Ethics number 1-10-72-131-15) and conducted according to the principles expressed in the Declaration of Helsinki. Every participant provided informed consent. This research was registered at ClinicalTrials.gov (NCT00999310, NCT02526628).

### Cognitive evaluation

As previously described [6], participants from cohort 1 underwent a comprehensive battery of standardized neuropsychological tests to assess their neurocognitive functions. In the present study, only domains and subtests in which males with KS and control males differed significantly were included. These tests included: processing speed (Trail Making A (TMT-A) and B (TMT-B) [23], and the Coding subtest (WAIS-C) of Wechsler Adult Intelligence Scale III (WAIS-III)) [24], working memory (Digit Span (WAIS-DS) and Letter-Number Sequencing (WAIS-LN) subtests of WAIS-III)), visual performance (Block Design (WAIS-BD) and Matrix Reasoning (WAIS-MR) subtests of WAIS-III), verbal memory and learning (total score of Rey Auditory Verbal Learning Test (RAVL-tot)), verbal fluency and comprehension (Sentence Repetition (SR)), verbal performance (Verbal fluency test (Fluency), Similarities (WAIS-S) and Vocabulary (WAIS-V) subtests of WAIS-III), executive function (cards administered of the Wisconsin Card Sorting Test (WCST-trai) [25], total move score (TOL-tms) and total correct score (TOL-tcs) of the Tower of London) [26], and FSIQ, VIQ and PIQ. FSIQ, VIQ and PIQ was determined by regression equations derived from summary statistics data (e.g. mean, standard deviation, correlations of the subtest) from the WAIS-III Danish reference material [24]:

$${{{\rm{FSIQ}}}} = \, 	40.21+(1.13\times {{{\rm{WAIS}}}}{\mbox{-}}{{{\rm{S}}}})+(1.38\times {{{\rm{WAIS}}}}{\mbox{-}}{{{\rm{MR}}}}) \\ 	+ (2.10\times {{{\rm{WAIS}}}}{\mbox{-}}{{{\rm{V}}}})+(1.35\times {{{\rm{WAIS}}}}{\mbox{-}}{{{\rm{BD}}}});$$

$${{{\rm{VIQ}}}}=50.91+(2.14\times {{{\rm{WAIS}}}}{\mbox{-}}{{{\rm{S}}}})+(2.76\times {{{\rm{WAIS}}}}{\mbox{-}}{{{\rm{V}}}});$$

$${{{\rm{PIQ}}}}=49.86+(2.57\times {{{\rm{WAIS}}}}{\mbox{-}}{{{\rm{MR}}}})+(2.47\times {{{\rm{WAIS}}}}{\mbox{-}}{{{\rm{BD}}}}).$$

Participants from cohort 2 underwent a three-hour battery of standardized cognitive tests, designed to assess major cognitive functions. The tests were administered and scored by trained research assistants under the supervision of a licensed psychologist. The test battery consisted of Wechsler Adult Intelligence Scale IV (WAIS-IV) [27], Wechsler Memory Scale III (WMS–III) [28] and the Wisconsin Card Sorting Test (WCST) [25]. Only data from the WCST and the subtest of the WAIS that were also available for cohort 1 were included.

### DNA methylation and gene expression in blood

For cohort 1, all methylation data from peripheral blood were obtained from a previously published study [14]. For cohort 2, all methylation and RNA sequencing data from peripheral blood were obtained from a previously published study [15].

Raw intensity values for cohort 1 (Infinium HumanMethylation450k) and cohort 2 (Infinium MethylationEPIC) were imported and processed using the R package Minfi [29]. Cross-reactive probes and poorly performing probes, as indicated by a detection p-value < 0.01, were excluded from the analysis. The preprocess Funnorm normalization method [30] was applied to remove between-array variation inferred by control probes, followed by the conversion of methylation values to beta-values and M-values. For differential methylation analysis, M-values were analyzed using LIMMA [31], with differentially methylated positions defined as having an adjusted p-value < 0.05. Beta-values of the differentially methylated positions (DMPs) were used for downstream analysis.

For RNA expression, the fastq files underwent initial quality control using FastQC (Babraham Bioinformatics, Cambridge, UK). Adaptor removal and trimming of low-quality ends were performed using Trim Galore with default settings (Babraham Bioinformatics, Cambridge, UK). Transcript expression levels were quantified using Salmon [32], with a decoy-aware transcriptome index based on the hg38 transcriptome. Transcript abundances were summarized to the gene level using the R package Tximeta [33].

### Neural precursor cells (NPCs)

#### Derivation of human induced pluripotent stem cells (hiPSCs)

KS (47,XXY, n = 3) and control male (46,XY, n = 3) human induced pluripotent stem cell (hiPSC) lines were derived from amniocytes using non-integrating episomal vectors (Fig. 1) [34]. KS amniotic fluid-derived cell lines X4, X5, and X6 were obtained from the Coriell Institute, Camden, NJ, USA, under the identifiers GM02269, GM03091, and GM03535, respectively, and karyotypically normal control male amniocytes M7, M8, and M9 were obtained from Dr. Daniel L. Van Dyke at the Mayo Clinic, Rochester, MN, USA. Briefly, 1×10^6^ amniocytes from each line were reprogrammed via nucleofection with three non-integrating plasmids overexpressing human SOX2 and KL4 (pCXLE-hSK, plasmid #27078, Addgene, Watertown, MA, USA), human L-MYC and LIN28 (pCXLE-hUL, plasmid #27080, Addgene, Watertown, MA, USA), and human OCT3/4 and shRNA against p53 (pCXLE-OCT3/4-shp53, plasmid #27077, Addgene, Watertown, MA, USA) and plated onto gelatinized plates in DMEM media containing 10% FBS and 1% penicillin-streptomycin (Gibco, Grand Island, NY, USA). Following six-seven days in culture, cells were re-plated at single-cell density onto mouse embryo fibroblast (MEF) feeders in F12 media consisting of DMEM/F12, 20% Knockout Serum Replacer, 1x non-essential amino acids, 1x Glutamax-100, β-ME, and 1% penicillin-streptomycin (all from Gibco, Grand Island, NY, USA), supplemented with the HDAC inhibitors SAHA and Sodium Butyrate (Sigma, St. Louis, MO, USA). By day 20–28, following the emergence of iPSC colonies, cells were switched to F12 media supplemented with FGF-2 (Gibco, Grand Island, NY, USA) and without HDAC inhibitors. Colonies with typical iPSC morphology were individually picked up, expanded, and passaged with dispase on MEF feeders in F12 media supplemented with FGF. The derived iPSC clones were tested for the expression of the key endogenous pluripotency factors such as OCT4 and NANOG, and for episomal/viral clearance by testing for lack of the vector containing transcripts as described in Okita K. et al. [34]. Prior to differentiation, iPSCs were adapted to feeder-less conditions by passaging to matrigel- or laminin-coated plates using accutase.

hiPSC clones were karyotyped for commonly duplicated or deleted genomic regions using hPSC Genetic Analysis qPCR kit (STEMCELL Technologies, Vancouver, BC, Canada). X chromosome aneuploidy was confirmed by FISH, XIST genomic DNA-qPCR, and CNV analysis of 850 K DNA methylation array data. RT-qPCR for XIST expression was performed to screen for X-chromosome inactivation (XCI) erosion. Only the XXY cell lines with levels of *XIST* expression comparable to XX lines were used for differentiation and downstream molecular studies.

#### Differentiation of hiPSCs to neural precursor cells (NPCs)

hiPSCs were differentiated into neural precursor cells (NPCs) using a dual SMAD inhibition protocol adapted from previously published studies (Fig. 1) [35, 36]. Briefly, cells were densely plated into 24- or 12-well plates pre-coated with laminin-521 (BioLamina, Sundbyberg, Sweden). NPC differentiation was induced in BNMM media, consisting of a 1:1 mix of DMEM/F12 and neural basal media, supplemented with N2, B27, non-essential amino acids, Glutamax-100, Insulin-transferrin-selenium-sodium pyruvate (ITSA), β-ME, 1% penicillin-streptomycin (all from Gibco, Grand Island, NY, USA), and the SMAD inhibitors SB431542 and LDN193189 (Selleck, Houston, TX, USA). Following nine days of neural induction, cells were treated with Versene (Gibco, Grand Island, NY, USA) and collected using a cell scraper. After gentle trituration, cells were passaged into 12-well plates pre-coated with laminin-521 and fed with BNMM media without SMAD inhibitors for three days. Following the emergence of neural rosettes, cells were fed with BNMM media supplemented with FGF (Gibco, Grand Island, NY, USA) without SMAD inhibitors. Cell pellets were collected for downstream molecular analyses at day 19 of differentiation.

### DNA methylation and gene expression in neuro precursor cells

#### DNA extraction and methylation analysis in NPCs

DNA lysates were prepared using the DNeasy blood and tissue kit (Qiagen, Hilden, Germany). DNA from NPCs was subjected to bisulfite conversion using the EZ DNA Methylation kit according to the manufacturer’s protocol (Zymo Research, Irvine, CA, USA). Methylation data were generated using Illumina 850 K methylation arrays (Illumina, San Diego, CA, USA). Raw Idat files were read into R using the Bioconductor Minfi package and processed using the adjustedFunnorm function from the Bioconductor wateRmelon package. Briefly, this method first corrects for background binding and dye bias using NOOB [37], and then normalizes using functional normalization [30, 38], with an additional interpolation of the probes on the X and Y chromosomes to avoid sex bias. Probes with low binding (not significantly different from background) were excluded from further analyses.

#### RNA extraction, cDNA library preparation, and sequencing

NPCs were collected and stored in Qiazol (Qiagen, Hilden, Germany) before processing. Total RNA was isolated using the Qiagen RNeasy mini kit (Qiagen, Hilden, Germany). Bulk RNA-seq indexed libraries were prepared using Illumina TruSeq RNA sample preparation kit V2 (Illumina, San Diego, CA, USA). All libraries were generated from 1 µg of total RNA starting material prior to mRNA isolation. cDNA libraries were constructed for analysis on a NextSeq sequencer and 75 bp single-end reads were generated.

#### RNAseq data analysis

The fastq files underwent initial quality control using FastQC (Babraham Bioinformatics, Cambridge, UK). Adaptor removal and trimming of low-quality ends were performed using Trim Galore with default settings (Babraham Bioinformatics, Cambridge, UK). Transcript expression levels were quantified using Salmon [32], with a decoy-aware transcriptome index based on the hg38 transcriptome. Transcript abundances were summarized to the gene level using the R package Tximeta [33].

### Weighted correlation network analysis

A weighted gene correlation network analysis (WGCNA, v1.70.3) [39] was applied to methylation data from cohort 1. The analysis was restricted to males with KS, evaluating the potential link between methylation patterns and neurocognitive traits within the KS group.

The beta-values of the differentially methylated positions (DMPs) between KS and controls (n_DMPs_ = 32 977) were used as input for the analysis. A signed co-expression network was constructed using a one-step approach, calculating adjacency by choosing an appropriate soft thresholding power with approximate scale-free topology. Clustering was performed on the signed Topology Overlap Matrix by hierarchical clustering, identifying modules via the blockwiseModules function. The module eigengenes were calculated via the moduleEigengenes function, and eigengene significance and corresponding p-value were obtained for each module-trait association.

### Combined methylation score of *TRAK1* and multiple linear regression including ENST00000341421.7

To create a combined methylation score, principal component analysis (PCA) was performed on the methylation levels of the CG-sites that were highly correlated to FSIQ in cohort 2. The first principal component (PC1) was used as the combined methylation score, capturing the largest variance in the methylation data. Multiple linear regression analysis was then conducted using the combined methylation score and ENST00000341421.7 expression as predictors, and with FSIQ as the dependent variable.

### Protein sequence alignment analysis

We obtained the amino acid sequence of full-length TRAK1 (Transcript ID: ENST00000327628.10) and TRAK1 isoform 686 (Transcript ID: ENST00000341421.7) from Ensembl [40]. The sequence alignment analysis was conducted using Clustal Omega (version 1.2.4, European Bioinformatics Institute, Hinxton, UK) with default parameters [41].

### Statistical analyses

Two-sample comparisons were performed using Welch T-tests for parametric and Mann-Whitney U-tests for non-parametric data. Linear regressions were performed using Pearson correlations for parametric and Spearman correlations for non-parametric data. Normality was evaluated using Quantile-Quantile plots. Equality of variance between groups was tested with Levene’s test. P < 0.05 was used as the significant threshold. Data was presented as boxplots showing the median and spread (IQR). Graphs were drawn in R (R Foundation for Statistical Computing, Vienna, Austria) and BioRender (BioRender, Toronto, ON, Canada).

## Results

### Weighted gene correlation network analysis (WGCNA) revealed potential links between DNA methylation patterns and neurocognitive traits in KS

**Fig. 2:**
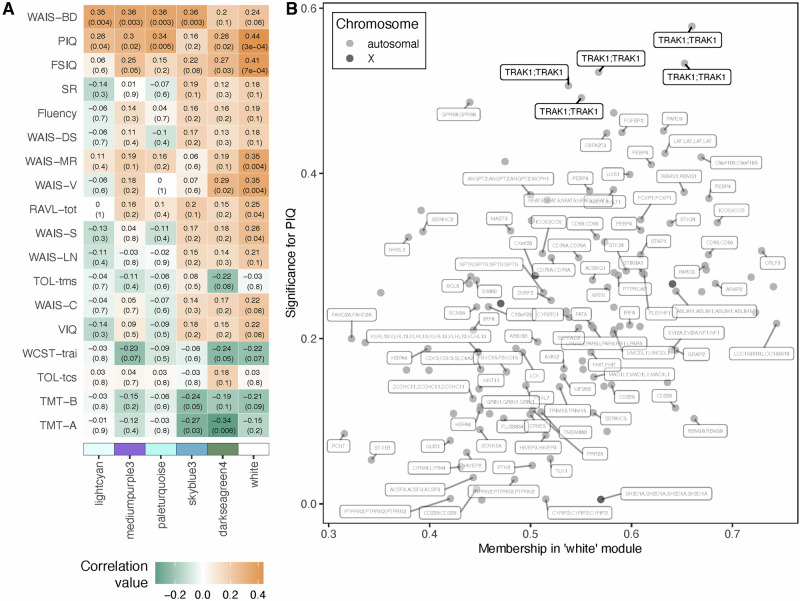
Weighted gene correlation network analysis (WGCNA). A WGCNA was performed on DNA methylation data from peripheral blood samples of individuals with KS (47,XXY) from cohort 1, to construct modules of CG-sites with similar methylation patterns and correlate these with neurocognitive traits. **A** Module-trait correlation plot. Color indicates correlation value, with negative correlations marked in green and positive correlations in orange. Numbers in each square indicate correlation values (top) and p-values (bottom, in parentheses). Only module-trait associations containing significant correlations are shown. The complete plot can be found in Supplementary fig. 1. **B** Module membership and trait significance. Scatterplot displaying the membership within the “white” module and significance for performance IQ (PIQ) for each CG-site within the “white” module. Dark points indicate X-chromosomal CG-sites whereas light points indicate autosomal CG-sites. Labels indicate UCSC gene name(s) for each CG-site, if available. Five CG-sites within the *TRAK1* gene with high significance for PIQ are highlighted.

**Fig. 3:**
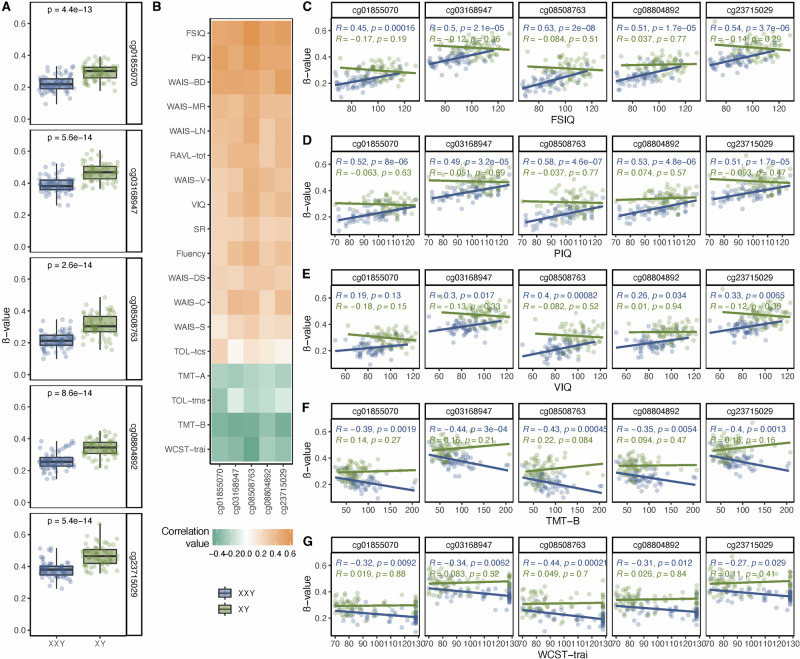
Methylation at CG-sites within *TRAK1* and correlation to neurocognitive traits. **A** ß-values for the five *TRAK1* CG-sites from the “white” module, comparing methylation levels in peripheral blood samples from males with KS (XXY) and male controls (XY). **B** Correlation values between the five *TRAK1* CG-sites from the “white” module and neurocognitive traits. Color indicates correlation value, with negative correlations marked in green and positive correlations in orange. **C-G** Correlations between the five *TRAK1* CG-sites from the “white” module within peripheral blood samples of males with KS (XXY) and male controls (XY), and full scale IQ (FSIQ,**C**), performance IQ (PIQ, **D**), verbal IQ (VIQ, **E**), Trail Making B (TMT-B, **F**) and Wisconsin Card Sorting Test (WCST-trai, **G**).

To investigate potential links between DNA methylation patterns and neurocognitive traits in KS, we performed WGCNA on methylation data from peripheral blood samples. DMPs between KS and male controls from cohort 1 were used as input. This initial analysis was restricted to males with KS from cohort 1 to evaluate the potential link between methylation patterns and neurocognitive traits within the KS group (Fig. 2A: Supplementary fig. 1: Table 1). Here, we examined correlations between the collective methylation status of co-methylated positions, termed modules, and neurocognitive traits. One module correlated with several neurocognitive traits (“white”), indicating that this set of CG-sites could be related to general neurocognitive function in KS. The strongest correlation was found to PIQ (r = 0.44, p = 3e–04). To further pinpoint which CG-sites within the “white” module had the strongest significance for PIQ, we calculated at the module-membership of the CG-sites within the “white” module in relation to significance for PIQ (Fig. 2B). Interestingly, five CG-sites within the *TRAK1* gene had both high module-membership and high significance for PIQ. The five identified CG-sites within the *TRAK1* gene from the “white” module were identified as cg01855070, cg03168947, cg08508763, cg08804892, and cg23715029. To further explore these five CG-sites from the “white” module, we analyzed the methylation levels at these sites in peripheral blood samples from cohort 1. Our analysis revealed that the five CG-sites were hypomethylated in males with KS compared to male controls (Fig. 3A).

Our WGCNA analysis did identify methylation patterns of CG-sites within other modules, exhibiting high module-membership and significance for neurocognitive traits (Supplementary Table 1). However, the correlation between other modules and neurocognitive traits were not as strong as for the “white” module. Therefore, we focused on the five CG-sites within the *TRAK1* gene throughout the remainder of this article.

### The methylation level at five CG-sites within *TRAK1* correlated specifically with neurocognitive performance in KS, and not in male controls

To examine the relationship between the methylation level at the five identified CG-sites within the *TRAK1* gene and the neurocognitive traits measured in males with KS, we performed additional correlation analyses (Fig. 3B-G: Supplementary fig. 2). The methylation levels at the five CG-sites were strongly correlated with the majority of neurocognitive traits in KS, with the strongest correlation observed for FSIQ (cg01855070: r = 0.45, p = 0.000160; cg03168947: r = 0.50, p = 2.1e–05; cg08508763: r = 0.63, p = 2e–08; cg08804892: r = 0.51, p = 1.7e–05; cg23715029: r = 0.54, p = 3.7e–06) and PIQ (cg01855070: r = 0.52, p = 8e–06; cg03168947: r = 0.49, p = 3.2e–05; cg08508763: r = 0.58, p = 4.6e–07; cg08804892: r = 0.53, p = 4.8e–06; cg23715029: r = 0.51, p = 1.73e–05). Positive correlations were observed for most neurocognitive traits, and negative correlations for TMT-A, TMT-B, TOL-tms, and WCST-trai. This was expected, since a lower score indicates better performance for these latter four variables. To determine whether the correlation between the methylation level at the five *TRAK1* CG-sites and the neurocognitive traits was specific to KS, we also investigated the correlations between the CG-sites and the neurocognitive traits in male controls from cohort 1 (Fig. 3C-G: Supplementary fig. 2). No significant correlations were found in male controls, indicating that the correlation between the methylation levels at the five CG-sites within *TRAK1* and neurocognitive performance is specific to KS.

### The methylation pattern of the five CG-sites within *TRAK1* in KS are consistent across cohorts

**Fig. 4:**
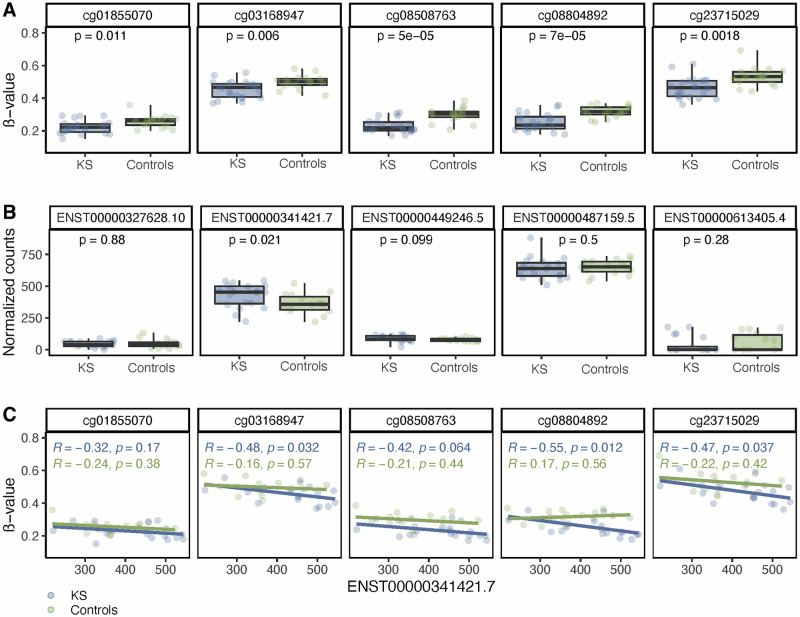
Methylation at CG-sites within *TRAK1* and expression of *TRAK1* transcripts. **A** ß-values for the five *TRAK1* CG-sites from the “white” module, comparing methylation levels in peripheral blood samples from males with KS (XXY) and controls (XY) from cohort 2. **B** Expression of *TRAK1* transcripts, comparing expression levels in peripheral blood samples from males with KS (XXY) and controls (XY) from cohort 2. **C** Scatter plot of ß-values for the five *TRAK1* CG-sites from the “white” module and expression of ENST00000341421.7 (st-TRAK1) in peripheral blood samples from males with KS (XXY) and controls (XY) from cohort 2.

We next validated our findings from cohort 1 by assessing the methylation level at the five identified CG-sites within *TRAK1* in peripheral blood samples from a second cohort (cohort 2). The analysis of peripheral blood samples from cohort 2 showed the same methylation pattern at the five CG-sites with hypomethylation in KS compared to male controls (Fig. 4A).

### The expression level of a short *TRAK1* transcript correlated negatively with the methylation level at the five CG-sites within *TRAK1* in KS

To investigate the transcriptomic effect of the altered methylation signature at the five identified CG-sites within *TRAK1*, we analyzed *TRAK1* expression in peripheral blood samples from cohort 2. No overall difference in *TRAK1* expression was detected between KS and controls (Supplementary fig. 3). However, analysis of the different *TRAK1* transcripts revealed that transcript ENST00000341421.7 (hereafter referred to as (short transcript *TRAK1* (st-TRAK1)) was upregulated in KS compared to controls (Fig. 4B). No difference in the expression of other *TRAK1* transcripts was observed between the two groups in cohort 2. We then examined the relationship between the expression of st-TRAK1 and the methylation level at the five identified CG-sites within *TRAK1*. Interestingly, the methylation level at these CG-sites was negatively correlated with the expression level of st-TRAK1 in KS, but not in controls (Fig. 4C). This agreed with the location of the five CG-sites, which are positioned upstream of the transcription start site (TSS) of st-TRAK1 (Supplementary fig. 4). Specifically, cg01855070, cg08508763, and cg08804892 were within 1500 bp upstream of the TSS (TSS1500) while cg03168947 and cg23715029 were within 200 bp upstream of TSS (TSS200). Thus, the five CG-sites may influence the promoter activity of st-TRAK1.

To further validate our findings from cohort 1, we investigated the correlation between the five CG-sites within *TRAK1* and the neurocognitive traits in cohort 2 (Table 2). Despite the smaller number of individuals in cohort 2 compared to cohort 1, we found that these CG-sites correlated with several neurocognitive traits in KS (Supplementary fig. 5). The strongest correlations were again observed for FSIQ (cg01855070: r = 0.2, p = 0.37; cg03168947: r = 0.54, p = 0.0093; cg08508763: r = 0.4, p = 0.068; cg08804892: r = 0.48, p = 0.025; cg23715029: r = 0.52, p = 0.014) and PIQ (cg01855070: r = 0.27, p = 0.22; cg03168947: r = 0.48, p = 0.024; cg08508763: r = 0.4, p = 0.064; cg08804892: r = 0.57, p = 0.0055; cg23715029: r = 0.53 p = 0.012).

As described above, four of the five CG-sites within *TRAK1* were highly correlated with FSIQ in cohort 2 (cg08508763, cg23715029, cg03168947, cg08804892). As gene expression data (RNAseq) was also present for all KS individuals in cohort 2, we created a combined methylation score for these four CG-sites to capture the overall methylation pattern. This was done using principal component analysis (PCA). Component 1 (PC1) was used as the combined methylation score and was then included in a linear multiple regression model along with st-TRAK1 expression to assess their joint effect on FSIQ. The model was significant (p = 0.014), indicating that the combined predictors significantly contribute to explaining the variation in FSIQ. Similarly, when PIQ and VIQ were tested as outcome variables in our multiple linear regression analysis, significant associations were observed for both PIQ (p = 0.048) and VIQ (p = 0.045). Furthermore, the combined model indicates that while st-TRAK1 alone is not a strong predictor of FSIQ, its interaction with the combined methylation pattern might be relevant for cognitive outcomes.

### DNA methylation and neurocognitive functions were stable over time

Eleven of the males with KS were included in both cohort 1 and cohort 2 and therefore had blood samples taken for DNA methylation analysis and underwent cognitive testing at two different time points. This enabled us to investigate whether the methylation levels measured by two different arrays (Infinium HumanMethylation450k and Infinium MethylationEPIC) were consistent and stable over time. Our data revealed a high degree of concordance across the five CG-sites (Supplementary fig. 6A). Furthermore, neurocognitive performance also revealed a high level of concordance over time (Supplementary fig. 6B).

### Hypomethylation at the five CG-sites within *TRAK1* and upregulation of the short *TRAK1* transcript observed in 47,XXY neural precursor cells

**Fig. 5:**
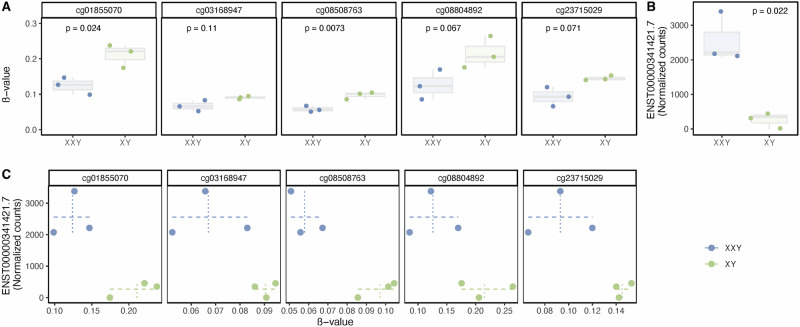
Methylation at CG-sites within *TRAK1* and expression of ENST00000341421.7 (st-TRAK1) within neural precursors. **A** ß-values for the five *TRAK1* CG-sites from the “white” module, comparing methylation levels in XXY and XY neural precursors. **B** Expression of ENST00000341421.7 (st-TRAK1) in XXY and XY neural precursors. **C** Scatter plot of ß-values for the five *TRAK1* CG-sites from the “white” module and expression of ENST00000341421.7 (st-TRAK1) in XXY and XY neural precursors. Solid and dotted lines indicate the range of methylation and expression, respectively, within the karyotype groups.

In the neurocognitive phenotype of KS, the brain is the primary target organ of interest. Due to the inaccessibility of brain samples from males with KS, we used neural precursor cells differentiated from human induced pluripotent stem cells derived from amniotic fluid cells with either a 47,XXY or a 46,XY karyotype. This confirmed that the five CG-sites within *TRAK1* were hypomethylated in neural cells with a 47,XXY karyotype compared to neural cells with a 46,XY karyotype (Fig. 5A). Additionally, we investigated the expression level of the st-TRAK1 in the neural precursor cells. In 47,XXY cells, st-TRAK1 was highly upregulated compared to 46,XY cells (Fig. 5B). No difference in the expression of other *TRAK1* transcripts was observed (Supplementary fig. 7). Next, we analyzed the relation between the methylation level at the five CG-sites within *TRAK1* and the expression level of st-TRAK1. For all CG-sites, we found a clear pattern between hypomethylation and higher expression levels (Fig. 5C). Thus, the differences observed in blood cells were also observed in neuronal cells.

### The short *TRAK1* transcript encodes a 686-amino acid isoform of TRAK1 that lacks segments from both the N-terminal and C-terminal regions compared to full-length TRAK1

The *TRAK1* gene encodes trafficking kinesin protein 1 (TRAK1), a 953-amino acid long protein [42]. St-TRAK1 encodes a 686-amino acid isoform of TRAK1 (hereafter referred to as TRAK1 isoform 686) [40]. To compare TRAK1 isoform 686 with full-length TRAK1, we performed a protein sequence alignment analysis. The analysis showed that most of the protein sequence was identical except that TRAK1 isoform 686 is missing segments from both the N-terminal and C-terminal (Supplementary fig. 8), suggesting that the full-length TRAK1 and TRAK1 isoform 686 may have functional differences.

## Discussion

Here, we present an integrated analysis of the neurocognitive profile, the methylome, and the transcriptome in peripheral blood samples and neuronal precursor cells from males with KS and male controls. We identified correlations, exclusively in KS, between the methylation level at five specific CG-sites (cg01855070, cg03168947, cg08508763, cg08804892, cg23715029) within *TRAK1* and several neurocognitive traits, including FSIQ, VIQ, and PIQ. Furthermore, we demonstrated a significant correlation between the expression level of st-TRAK1 and the methylation level at the five CG-sites within *TRAK1* in KS. The five CG-sites within *TRAK1* are located upstream of the TSS of the st-TRAK1, suggesting that they control its expression. In neural precursor cells derived from 47,XXY amniotic cells, we similarly observed hypomethylation at these CG-sites and highly increased expression of st-TRAK1 compared to control cells. To our knowledge, this is the first study linking a specific methylation signature to the neurocognitive phenotype in KS.

**Fig. 6:**
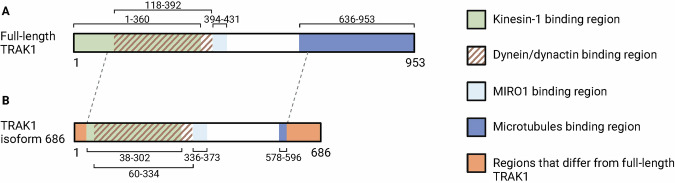
Diagram of the full-length TRAK1 protein and the TRAK1 isoform 686 protein. **A** Diagram of the full-length TRAK1 protein with important regions for mitochondrial transport. The kinesin-1 binding region (residues within 1–360) is shown in green, the dynein/dynactin binding region (118–392 residues) is depicted with stripes, the MIRO1 binding region (394–431 residues) is shown in light blue, and the microtubules binding region (636–953 residues) is shown in dark blue. **B** Diagram of the TRAK1 isoform 686 protein. Sequence alignment analysis has shown that TRAK1 isoform 686 contains the dynein/dynactin binding region (60–334 residues; striped), the MIRO1 binding region (336–373 residues; light blue), and only part of the binding region for kinesin-1 (38–302 residues; green) and microtubules (578–596 residues; dark blue). Dotted lines demonstrate where the sequence of the full-length TRAK1 and the TRAK1 isoform 686 is identical. Parts of the C-terminal and N-terminal region of TRAK isoform 686 (shown in orange) differ from the full-length. This figure was made using BioRender.com.

The *TRAK1* gene, mapping to 3p22.1, is widely expressed, including in the fetal and adult brain [43]. It encodes trafficking kinesin protein 1 (TRAK1), that is a 953-amino acid long protein with a molecular mass of 106 kDa (Fig. 6A) [42]. *TRAK1* variants and single nucleotide variants have been associated with a wide range of neurological and neuropsychological disorders/conditions including schizophrenia [44], autism spectrum disorders [45–47], trigeminal neuralgia [48], encephalopathy [49], seizures, epilepsy, developmental delay [47, 49–54], as well as anatomical brain alterations, including brain atrophy [49–52]. The broad spectrum of neurological and neuropsychological disorders/conditions linked to variants in *TRAK1* suggests that *TRAK1* is crucial for normal neuronal cell function. Recently, an association between the methylation level at one CG-site near the *TRAK1* gene (not one of the five CG-sites found in this study) and the cognitive function in monozygotic twins with normal karyotype was demonstrated [55], further supporting that altered methylation at specific CG-site in relation to *TRAK1* might be related to neurocognitive performance.

Several studies have demonstrated that *TRAK1* plays an essential role in mitochondrial mobility. In neurons, the TRAK1 protein is abundantly localized in axons to facilitate the axonal transport of mitochondria along microtubules by acting as an adaptor protein that links mitochondria to kinesin-1 (for anterograde transport) and dynein/dynactin (for retrograde transport) [56]. Knockdown of *TRAK1* in neurons significantly reduces the mitochondrial motility in the axons, highlighting its importance for proper axonal transport of mitochondria [56, 57]. Moreover, the mitochondrial distribution and motility are disrupted in cells carrying a pathogenic *TRAK1* variant [49]. In neurons, mitochondria are crucial for growth, survival, and function, including initiation of action potentials and synaptic transmission, by producing ATP and buffering intracellular Ca^2+^ [58]. Mitochondrial transport within neurons is not only responsible for distributing mitochondria to distal regions like synapses to meet local energy demands but also for removing and replacing aged and dysfunctional mitochondria with healthy ones [58]. Defects in mitochondrial transport are linked to several neurodegenerative diseases including Alzheimer’s disease [59].

The N-terminal domain of TRAK1 is essential for both the kinesin-1 and dynein/dynactin-dependent transport (Fig. 6A) [56, 60]. Based on comparisons with the cryo-EM structures of other adaptors that interact with dynein/dynactin, it is predicted that the region of TRAK1 implicated in binding dynein/dynactin spans amino acids 118 to 392 [61]. This region of TRAK1 contains the CC1 box and the Spindly motif that bind dynein light intermediate chain and the pointed-end complex of dynactin, respectively [60, 62, 63]. The exact binding site of TRAK1 for kinesin-1 is unknown but appears to involve residues within 1–360 [56, 60, 64]. Co-immunoprecipitation experiments further revealed that residues 100–360 of TRAK1 could efficiently bind to kinesin-1 [56], while a more recent study using crosslinking mass spectrometry demonstrated that residues 23–138 were essential for the interaction of TRAK1 with kinesin-1 [64]. TRAK1 interacts with the mitochondria by binding MIRO proteins anchored to the mitochondrial outer membrane [65]. Just like TRAK1, MIRO proteins are crucial for proper mitochondrial transport, with MIRO1 identified as the main isoform involved in mitochondria trafficking in neurons [66]. Baltrusaitis et al. demonstrated that TRAK1 residues 394–431 are sufficient for binding MIRO1 [61]. The C-terminal region of TRAK1 (residues 636–953) also plays a role in mitochondrial trafficking by interacting with microtubules [67], which increases the processivity of the TRAK1-kinesin-1 complex, as shown by increased run length and microtubule interaction time of the complex for full-length TRAK1 compared to a TRAK1 mutant lacking its C-terminal [67].

In the present study, we demonstrated that most of the protein sequence of full-length TRAK1 and TRAK1 isoform 686 is identical. However, TRAK1 isoform 686 lacks segments from both the N-terminal and C-terminal (Fig. 6). Based on our protein sequence alignment analysis, TRAK1 isoform 686 contains binding sites for dynein/dynactin and MIRO1, but only part of the binding site for kinesin-1, suggesting that its affinity for kinesin-1 may be reduced compared to full-length TRAK1 (Fig. 6B). Additionally, its truncated C-terminal may prevent or diminish its microtubule binding, leading to reduced processivity of the TRAK1-kinesin-1 complex, according to Henrichs et al [67]. Consequently, we hypothesize that TRAK1 isoform 686 may be less effective at transporting mitochondria compared to full-length TRAK1. The observed upregulation of st-TRAK1 in KS indicates a disruption in the balance between full-length TRAK1 and TRAK1 isoform 686, potentially leading to impaired mitochondrial distribution and motility in neurons in KS. This disruption may contribute to the neurocognitive phenotype observed in KS, although experimental validation will be necessary. Future experiments should investigate the impact of st-TRAK1 upregulation on mitochondria trafficking in KS and examine its effect on cognitive functions including memory in animal models.

Besides being involved in mitochondria trafficking, TRAK1 has been implicated in other biological processes, including regulation of endosomal trafficking [68] and the endocytic trafficking of the gamma-aminobutyric acid (GABA)-A receptor [69]. However, it is unclear whether these processes are affected by hypomethylation at the five CG-sites within *TRAK1* and upregulation of st-TRAK1 in KS. TRAK1 isoform 686 could also be involved in additional functions in neurons as well as in other cell types. Thus, the link between *TRAK1* and the neurocognitive profile in KS may be influenced by factors beyond its role in mitochondrial transport.

Our findings demonstrate a strong correlation between the “white” module and general cognitive measures such as FSIQ and PIQ. While these measures are broad and not the most specific neurocognitive traits associated with KS, prior research has established that individuals with KS consistently exhibit FSIQ scores approximately 10 points lower than controls [5, 6, 8–11], underscoring its relevance as a marker of cognitive differences. Notably, we also observed significant correlations between the “white” module and neurocognitive traits more characteristic of KS, such as verbal performance, and verbal memory and learning. These results suggest that the methylation pattern of CG-sites in the “white” module is implicated not only in general cognitive performance, but also in cognitive domains more directly associated with the KS phenotype. Overall, these findings highlight the potential role of TRAK1 in shaping both KS-specific and general cognitive outcomes, offering deeper insights into the genotype-phenotype correlations in this condition.

The relationship between hypomethylation at the *TRAK1* sites and the presence of an additional X chromosome in XXY remains unclear. However, our findings are consistent with our prior studies and the broader literature in that while hypermethylation is the predominant epigenetic signature observed in XXY, instances of hypomethylation are also evident [15]. Hypomethylation at specific loci, including *TRAK1*, may represent a nuanced and locus-specific response to the additional X chromosome. Such responses could arise from factors such as the disruption of chromatin remodeling, altered transcription factor binding, or differential regulatory influences associated with having an additional X chromosome. Moreover, while *TRAK1* hypomethylation may appear contradictory to the global hypermethylation trend, it may highlight a unique regulatory mechanism at this locus. The coexistence of hyper- and hypomethylated sites reflects the complexity of epigenetic regulation in XXY. Further research, including transcriptomic and chromatin accessibility studies, is warranted to elucidate the X-linked biological drivers underlying these specific methylation changes and their functional implications.

Our findings highlight methylation at an autosomal gene, *TRAK1*, as the primary correlate of neurocognitive outcomes in KS, despite the presence of five X-chromosomal CG-sites within the “white” module, three of which localize within known genes (*CXorf23/BCLAF3*, *SH2D1A*, *KLHL13*). The relative paucity of module-specific methylation on X linked genes underscores the unexpected prominence of an autosomal in this context. This raises the possibility that *TRAK1* may be subject to indirect regulation by X chromosome-encoded transcription factors or that epigenetic modifiers could be at play. Prior studies, including our own, have demonstrated that X chromosome dosage effects induce widespread transcriptomic and epigenomic alterations across the genome, suggesting that X linked regulatory elements may exert transacting effects on autosomal genes [13–15, 20–22]. However, whether such mechanisms contribute to *TRAK1* regulation remains unclear. Additionally, given the well-documented endocrine alterations in KS (reviewed in [2]), it is plausible that *TRAK1* expression and methylation may be influenced by perturbed androgen signaling, potentially through androgen receptor binding sites or other hormone-responsive elements. While no direct link between testosterone-related pathways and the *TRAK1* promoter region has been established, investigating such interactions may provide novel insights into the molecular underpinnings of KS-associated neurocognitive phenotypes. These findings collectively reinforce the broader relevance of our work within the framework of sex chromosome aneuploidy and highlight critical avenues for future research into the interplay between X-linked regulatory factors, hormonal signaling, and autosomal gene expression.

We demonstrate that in KS, DNA hypomethylation at specific CG-sites within *TRAK1* and increased expression of st-TRAK1 are associated with specific neurocognitive phenotypes, including lower FSIQ, VIQ and PIQ, indicating a potential epigenetic basis for the neurocognitive impairments observed in KS. These specific CG-sites may be useful as epigenetic biomarkers for determining the level of neurocognitive function in KS. However, further validation using functional assays will be necessary to clarify their impact on the neurocognitive traits in KS.

### Data access

Human data used in this study has previously been published and is available from the European Genome-phenome Archive (EGA) under the accession number EGAS00001002797 (cohort 1), EGAS00001006996 and EGAS00001007020 (cohort 2). Data from neural precursor cells are available upon request.

## Supplementary information

Supplementary file

Supplementary Table 1
