# Supplementary material for: DNA hypomethylation at specific CG-sites within TRAK1 is linked to the neurocognitive profile in Klinefelter syndrome
Source: Mol Psychiatry. 2025 Sep 30;31(3):1409–19. doi: 10.1038/s41380-025-03254-z (PMC12916290; doi:10.1038/s41380-025-03254-z)
Supplement: Supplementary file 1 — Supplementary file [file 41380_2025_3254_MOESM1_ESM.docx]

**Supplementary Information:**

DNA hypomethylation at specific CG-sites within *TRAK1* is linked to the neurocognitive profile in Klinefelter syndrome


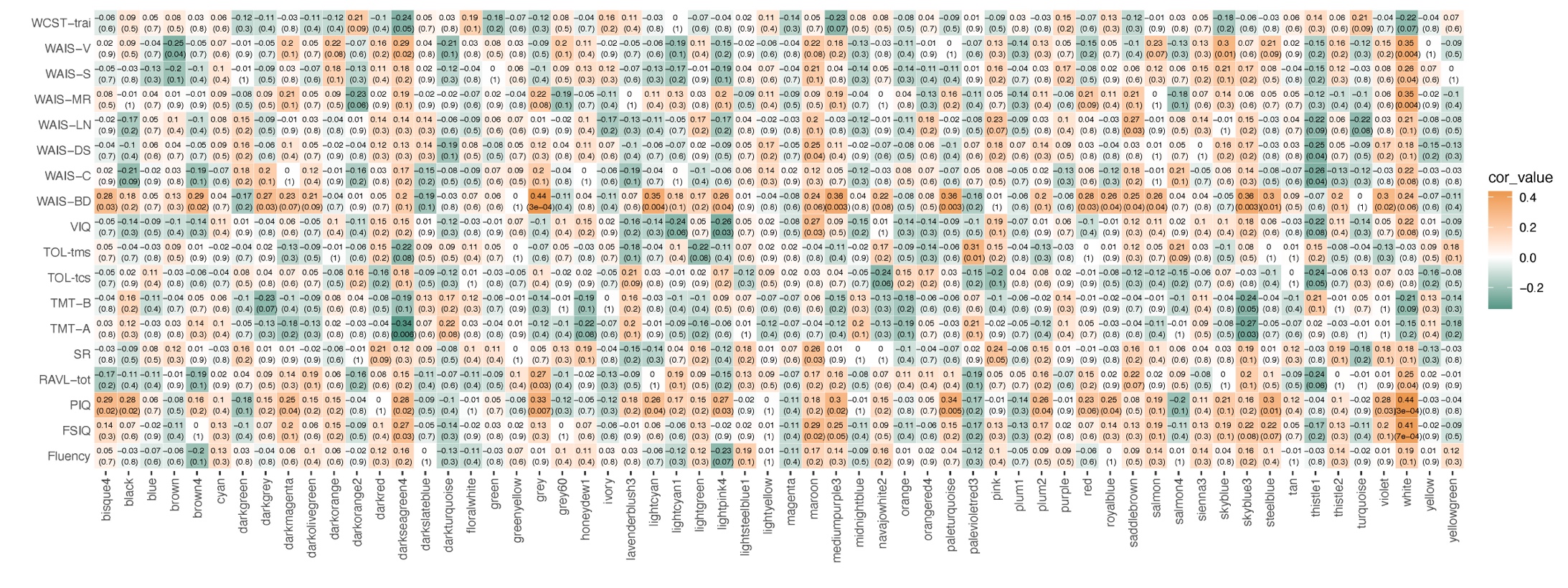


**Supplementary Figure 1**: Complete module-trait correlation plot.

A WGCNA was performed on methylation data from males with KS (47,XXY) from cohort 1 to construct modules of CG-sites with similar methylation patterns and correlate these to neurocognitive traits. Color indicates correlation value, with negative correlations marked in green and positive correlations in orange. Numbers in each square indicate correlation values (top) and p-values (bottom, in parentheses).


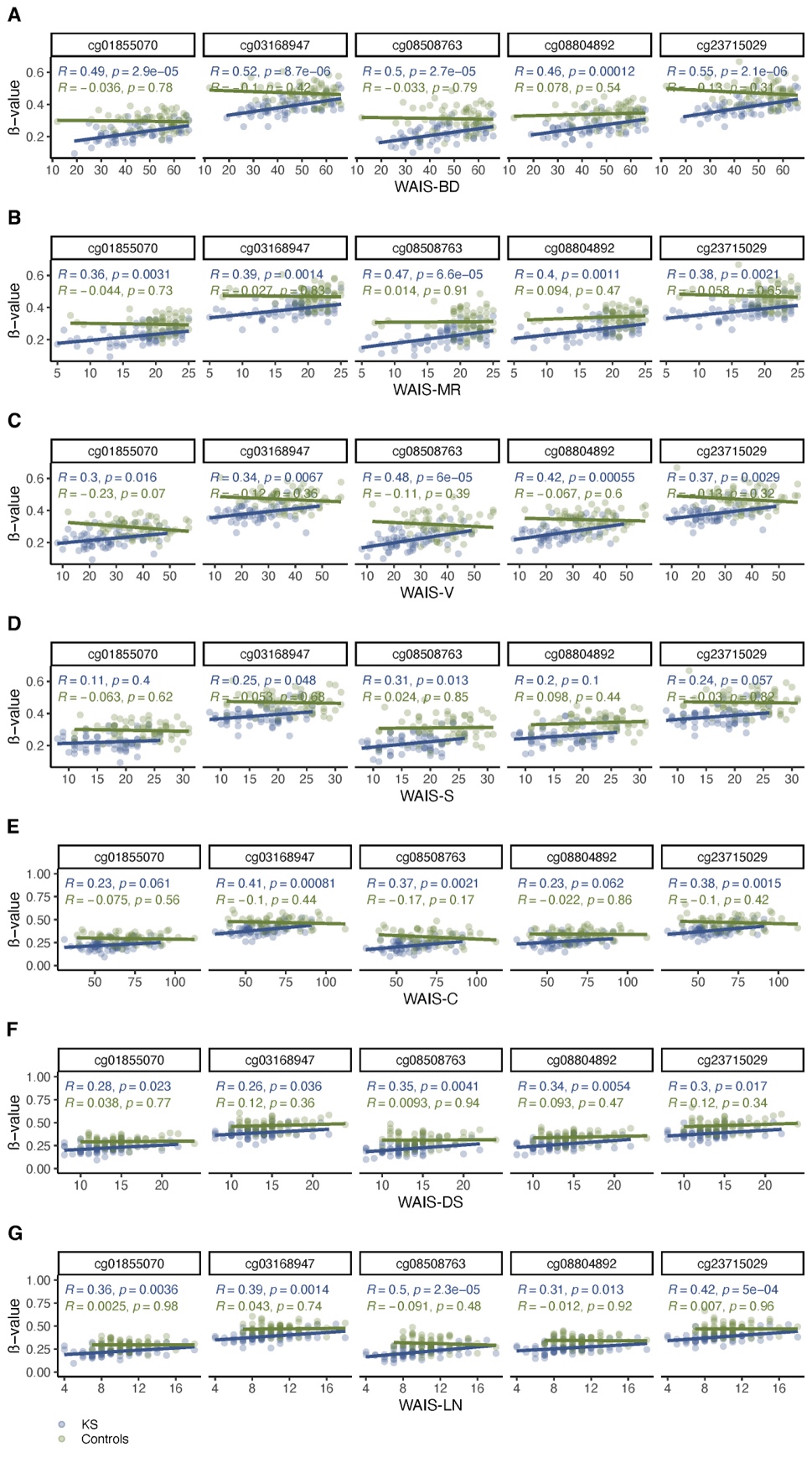


**Supplementary Figure 2**: Correlations between the five *TRAK1* CG-sites from the “white” module in peripheral blood samples from males with KS (XXY) and controls (XY) from cohort 1, and WAIS-BD (panel A), WAIS-MR(panel B), WAIS-V (panel C), WAIS-S (panel D), WAIS-C (panel E), WAIS-DS (panel F) and WAIS-LN (panel G).


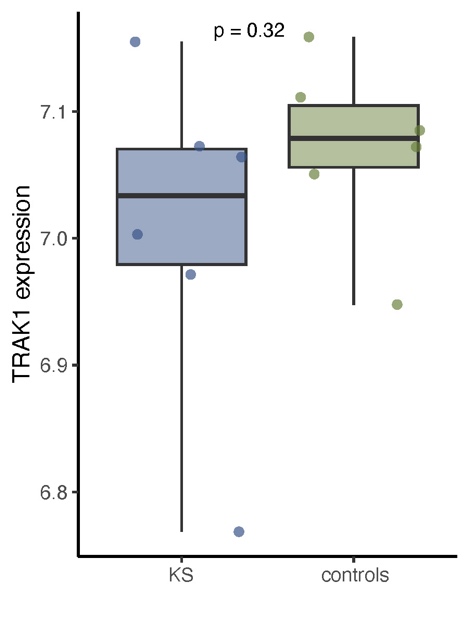


**Supplementary figure 3**: *TRAK1* expression in peripheral blood samples from males with KS (XXY) and controls (XY) from cohort 2.


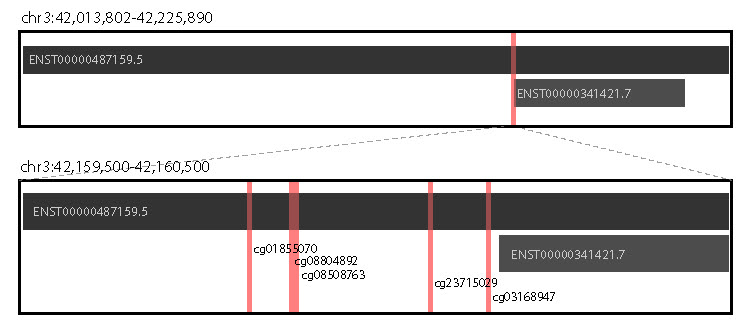


**Supplementary figure 4**: Localization of the five CG-sites within *TRAK1* (cg01855070, cg08804892, cg08508763, cg23715029, and cg03168947) in relation to *TRAK1* transcripts ENST00000487159.5 and ENST00000341421.7 (st-TRAK1).


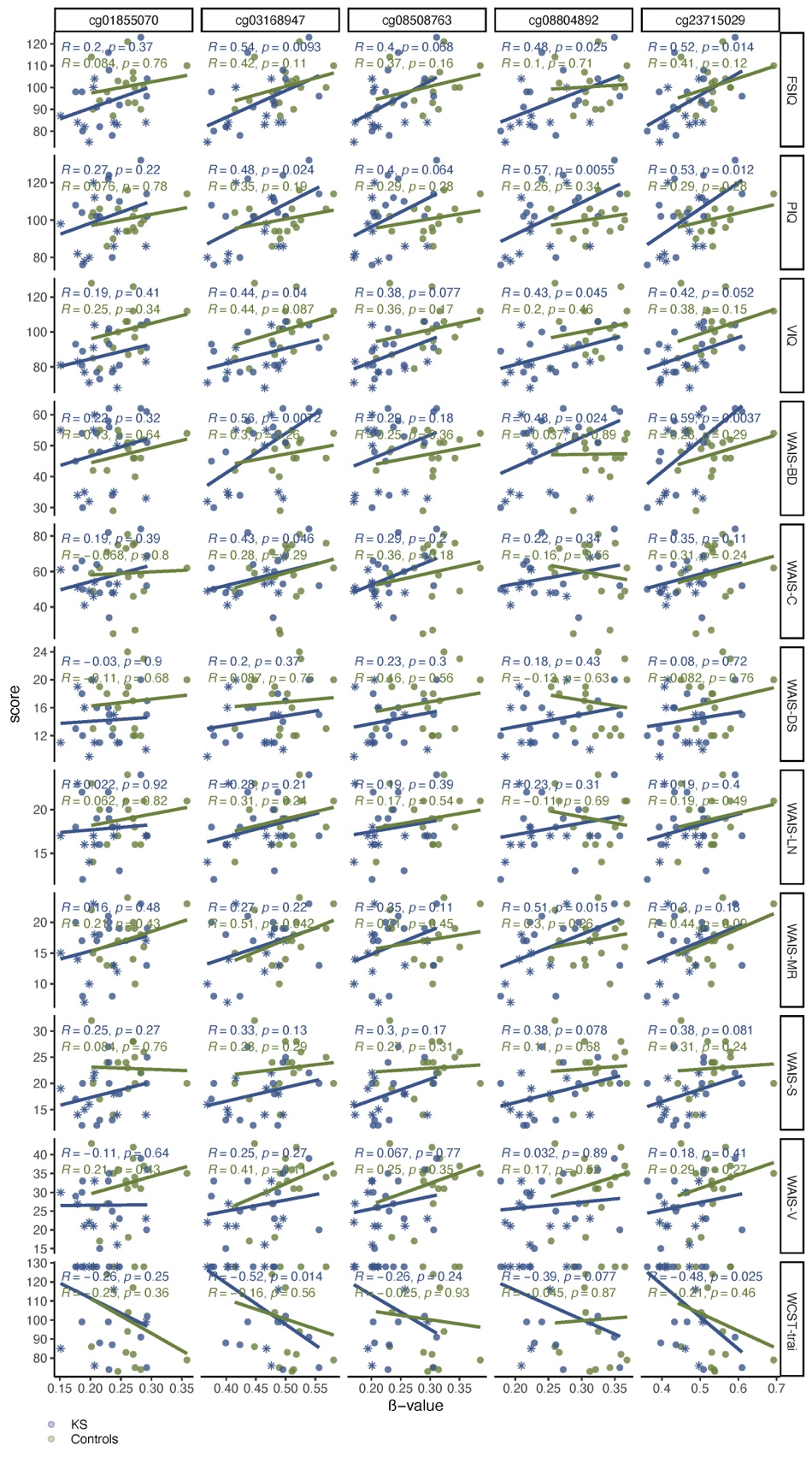


**Supplementary figure 5**: Correlations between the five *TRAK1* CG-sites from the “white” module in peripheral blood samples from males with KS (XXY) and controls (XY) from cohort 2, and full-scale IQ (FSIQ), performance IQ (PIQ), verbal IQ (VIQ) WAIS-BD, WAIS-C, WAIS-DS, WAIS-LN, WAIS-MR, WAIS-S, WAIS-V, and WAIS-trai.


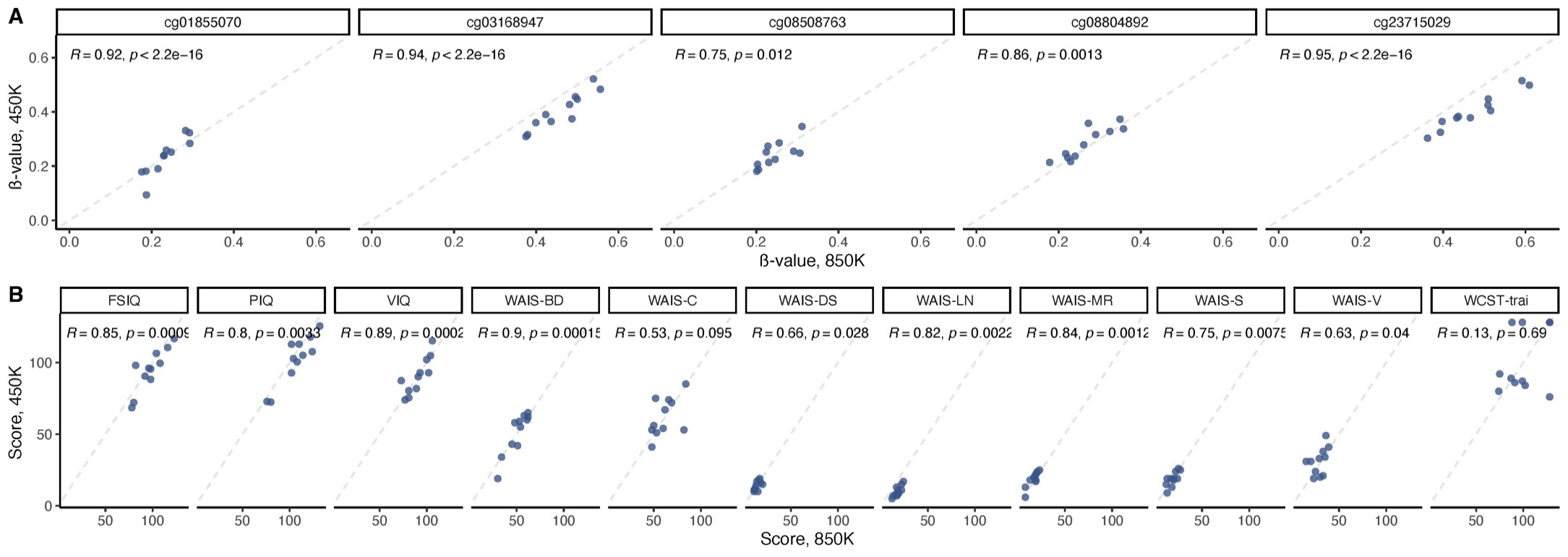


**Supplementary figure 6**: Correlation between methylation at the five *TRAK1* CG-sites from the “white” module in peripheral blood samples (A) and neurocognitive traits full-scale IQ (FSIQ), performance IQ (PIQ), verbal IQ (VIQ), WAIS-BD, WAIS-C, WAIS-DS, WAIS-LN, WAIS-MR, WAIS-S, WAIS-V, and WCST-trai (B) between males with KS appearing in both cohort 1 (y-axis; 450K) and cohort 2 (x-axis; 850K).


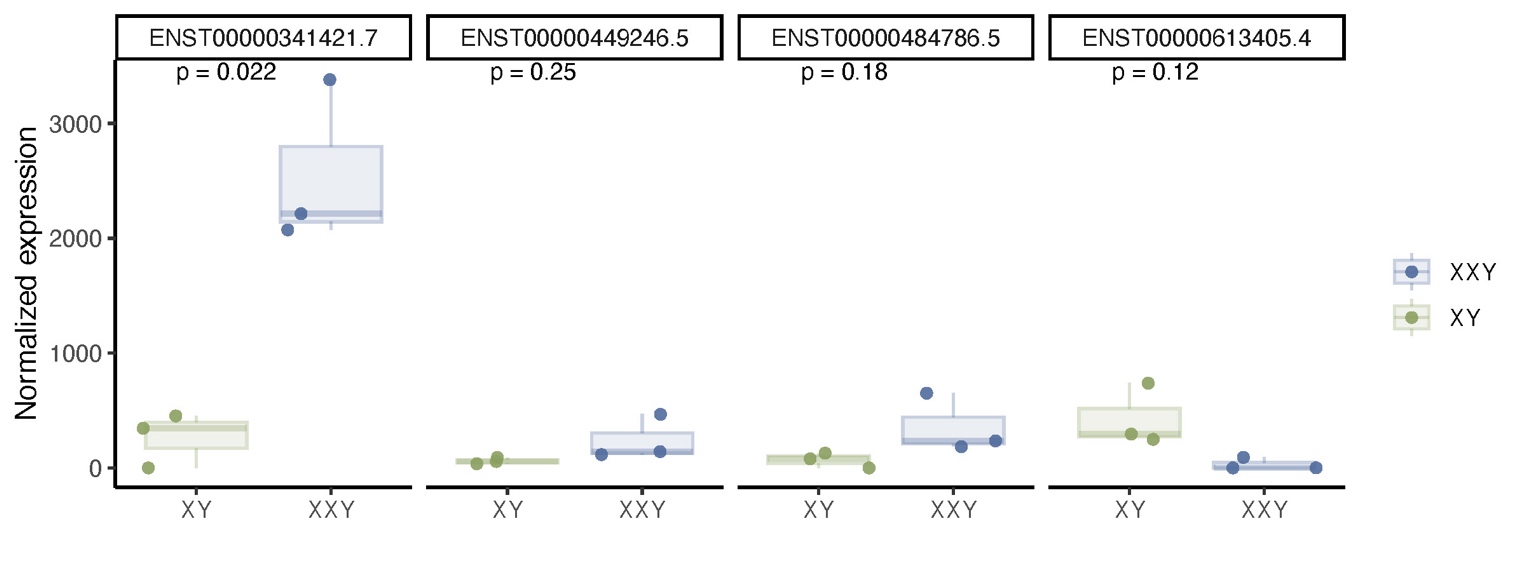


**Supplementary figure 7**: Expression of *TRAK1* transcripts in XXY and XY neural precursors.


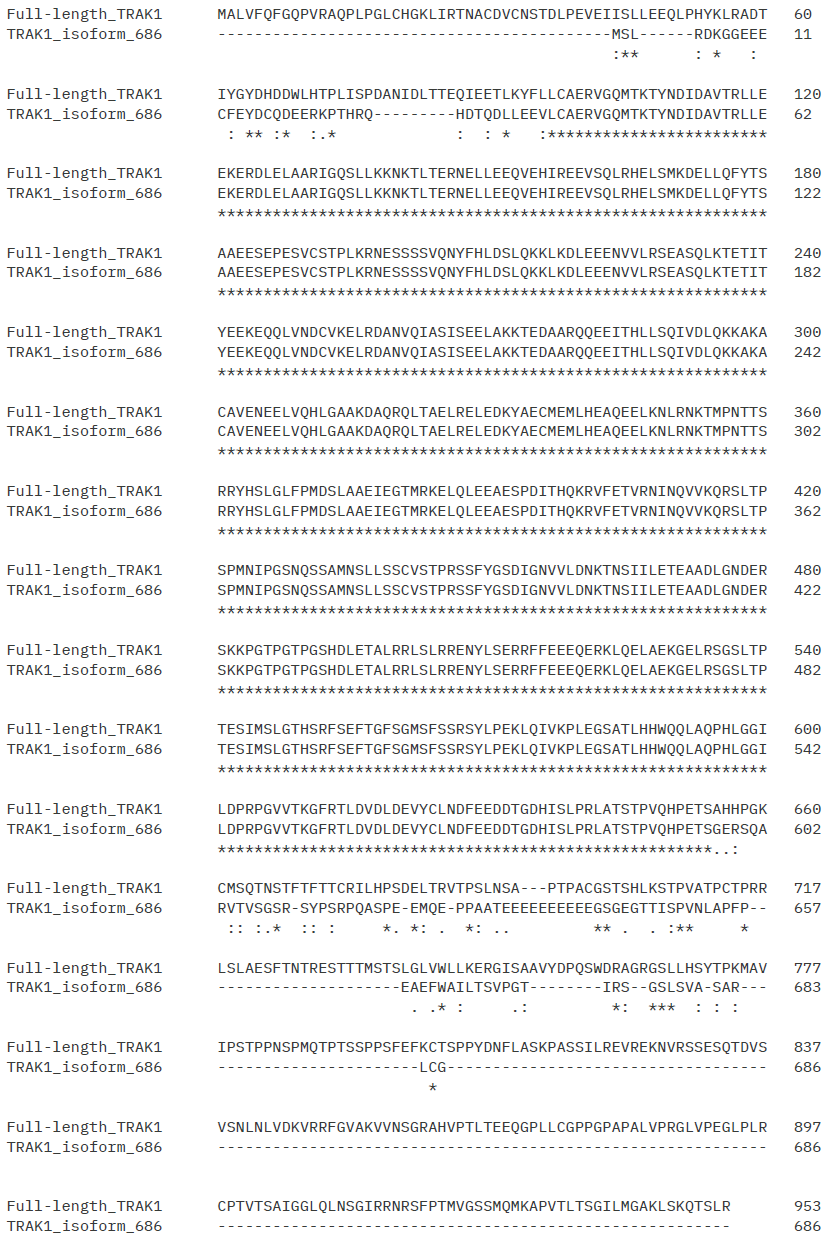


**Supplementary figure 8**: Sequence alignment analysis of full-length TRAK1 (with transcript ID: ENST00000327628.10) and TRAK isoform 686 (with transcript ID: ENST00000341421.7).

**Supplementary Table 1:** Module memberships and trait significances for CG-sites within selected modules obtained from WGCNA, and correlating traits (“white” module vs. PIQ, “darkseagreen4” module vs. TMT-A and “paleturquoise”, “skyblue3”, “lightcyan” and “mediumpurple3” modules vs. WAIS-BD). Module membership and trait significance values and p-values are provided, as well as chromosome, UCSC gene name and -group, if applicable.
